# Microarray analysis of lncRNA and mRNA expression profiles in patients with Legg-Calve-Perthes disease

**DOI:** 10.3389/fped.2022.974547

**Published:** 2022-09-07

**Authors:** Shangyu Wang, Haobo Zhong, Renhao Ze, Pan Hong, Jin Li, Xin Tang

**Affiliations:** ^1^Department of Orthopedics, Union Hospital, Tongji Medical College, Huazhong University of Science and Technology, Wuhan, China; ^2^Department of Orthopedics, Huizhou First Hospital, Huizhou, China

**Keywords:** Legg-Calve-Perthes disease, mRNA, lncRNA, vascular, networks

## Abstract

**Background:**

The etiology and underlying pathogenic mechanisms of Legg-Calve-Perthes disease (LCPD) still remain unclear. A disruption of blood supply to the femoral head, producing ischemic necrosis, appears to be the critical pathological event. The lncRNAs play crucial roles in many biological processes and are dysregulated in various human diseases. However, its expression profiles and the potential regulatory roles in the development of LCPD have not been investigated.

**Methods:**

In this study, differentially expressed lncRNA and mRNA of Legg-Calve-Perthes disease patients were profiled. Several GO terms and pathways that play important roles in the regulation of vascular structure, function or coagulation were selected for further analysis. The lncRNA -mRNA interacting networks in LCPD tissues were constructed to identify novel potential targets for further investigation.

**Results:**

The microarray analysis revealed that 149 lncRNAs and 37 mRNAs were up-regulated, and 64 lncRNAs and 250 mRNAs were down-regulated in LCPD tissues. After filtering, we finally found 14 mRNAs and constructed an mRNA-lncRNA interacting network. Through the analysis of the interaction network, we finally found 13 differentially expressed lncRNAs, which may be implicated in the pathogenesis of LCPD. These mRNAs/lncRNAs were further validated with qRT-PCR.

**Conclusion:**

The findings of this study established a co-expression network of disease-related lncRNAs and mRNAs which screened out from the concerned G.O. terms and Pathways, which may provide new sights for future studies on molecular mechanisms of LCPD.

## Introduction

Legg-Calve-Perthes disease (LCPD) is an idiopathic avascular osteonecrosis of the femoral head in children between 2 and 12 years. The reported incidence of LCPD varies from 0.2 to 19.1 per 100,000, and the boys are affected 5 times more often than girls (1, 2). LCPD affects the articular cartilage, epiphysis, growth plate, and metaphysis, leading to a progressive deformity of the affected hip. If left untreated, it results in secondary degenerative osteoarthritis in later life. Since the first recognition of LCPD as a separate disease entity in 1910, numerous theories about the etiology of the disease have been proposed. However, none have been validated conclusively, and the underlying mechanisms and determinants of LCPD still remain unclear (2, 3). In-depth understanding and treatment of LCPD are still a great challenge; hence more pathogenetic investigations are needed.

Evidence from clinical findings (4–8) and animal studies (9–11) support the concept that the disruption of blood supply to the femoral head, producing ischemic necrosis, appears to be the critical pathological event. For this reason, many researchers tried to look for vascular etiology. Many factors that might lead to disruption of blood supply to the femoral head, such as thrombophilia or decreased fibrinolysis (12–14), vascular anomalies (15), soluble selectins (16), and endothelial nitric oxide synthase gene polymorphism (17), have been proposed to be linked to the pathogenesis of LCPD. Other biological factors reported to be associated with the etiology or pathogenesis of LCPD include the mutation of the COL2A1 gene (18), abnormality in the insulin-like growth factor 1 pathway (19), abnormal lipid metabolism (20), and the higher level of circulating leptin (21). These findings imply that the dysregulation of multiple biological processes may be involved in the pathogenesis and development of LCPD.

Most diseases are frequently associated with altered transcription patterns, including the aberrant level of protein-coding RNAs and dysregulation of non-coding RNAs (ncRNAs) (22). A vast majority of human genome transcripts are ncRNAs, including microRNAs (miRNAs), long non-coding RNAs (lncRNAs), circular RNAs (circRNAs), and so on (23, 24). Among them, lncRNAs are endogenous molecules consisting of more than 200 nucleotides in length and play critical roles in many biological processes, such as transcriptional regulation of protein-coding genes, genomic imprinting, and cell differentiation and development (25, 26). There is accumulating evidence that the lncRNAs are dysregulated in various human diseases, which is indicated by the aberrant expression of certain lncRNAs in a variety of disorders (27). Many lncRNAs have been regarded as diagnostic biomarkers or therapeutic targets (28). Recently, the lncRNA expression profiles were screened in bone marrow mesenchymal stem cells (BMSCs) from patients with steroid-induced osteonecrosis of femoral head (ONFH) (29, 30). The results of these studies indicate that the dysregulated lncRNAs are closely associated with increased adipogenic and decreased osteogenic differentiation of BMSCs during the development of steroid-induced ONFH (29, 30). However, the lncRNA expression profiles and the potential regulatory network in the development of LCPD have not been investigated. In this study, we aimed at profiling differentially expressed lncRNA and mRNA and constructing lncRNA-mRNA interacting networks in LCPD tissues to identify novel potential targets for a better understanding of the pathogenesis of LCPD. Some of the identified sets of lncRNA and mRNA were subsequently confirmed by quantitative reverse transcription-polymerase chain reaction (qRT-PCR) in periosteum samples of LCPD.

## Materials and methods

### Patients' samples

A total of 9 LCPD patients and 6 children with a closed proximal femoral fracture who underwent surgical treatment (subtrochanteric osteotomy for LCPD, and open reduction and internal fixation for proximal femoral fracture) were selected for microarray analysis as case and control, respectively, in this study (Table 1). Samples from another 3 LCPD patients and 3 similar control patients were used for qRT-PCR validation (Table 2). These patients were diagnosed by the radiographic and physical examination in Wuhan Union Hospital (Wuhan, China). Periosteum specimens (size: about 1 cm × 0.5 cm) were biopsied from the proximal femur (subtrochanteric) of each patient during surgery and transferred to liquid nitrogen immediately after being washed with saline. Informed consent was obtained from the patient's guardians. The ethical review board approved this study in the author's institution.

**Table 1:** The main clinical characteristics of the pediatric periosteum samples for Microarray.

**Sample number**	**Sex**	**Age (years)**	**Operation site**	**Diagnosis**	**Stage**
Patient 1	Male	9	Right	LCPD	Reossification
Patient 2	Female	6	Left	LCPD	Reossification
Patient 3	Male	8	Left	LCPD	Osteonecrosis
Patient 4	Male	10	Right	LCPD	Fragmentation
Patient 5	Male	7	Right	LCPD	Reossification
Patient 6	Male	8	Right	LCPD	Reossification
Patient 7	Male	9	Left	LCPD	Fragmentation
Patient 8	Male	6	Left	LCPD	Reossification
Patient 9	Female	5	Left	LCPD	Reossification
Ctrl 1	Male	9	Left	PFF	-
Ctrl 2	Male	6	Left	PFF	-
Ctrl 3	Male	14	Left	PFF	-
Ctrl 4	Male	9	Right	PFF	-
Ctrl 5	Male	7	Right	PFF	-
Ctrl 6	Male	9	Left	PFF	-

LCPD, Legg-Calve-Perthes disease; PFF, proximal femoral fracture.

**Table 2:** The main clinical characteristics of the pediatric periosteum samples for qRT-PCR.

**Sample number**	**Sex**	**Age (years)**	**Operation site**	**Diagnosis**
Patient 1	Male	8	Left	LCPD
Patient 2	Male	4	Left	LCPD
Patient 3	Female	10	Right	LCPD
Ctrl 1	Female	7	Right	PFF
Ctrl 2	Male	10	Left	PFF
Ctrl 3	Male	5	Left	PFF

LCPD, Legg-Calve-Perthes disease; PFF, proximal femoral fracture.

### Microarray assay and data analysis

The lncRNA and mRNA expression profiling analysis was performed by Genminix Informatics Ltd., Co. (Shanghai, China) using GeneChip™ Human Transcriptome Assay (HTA) 2.0 (Affymetrix, USA). Briefly, total RNA was separately extracted from each tissue sample using the RNeasyMini Kit (QIAGEN, Germany) and transcribed into double-stranded complementary DNA (cDNA). Then the cDNA was fragmented, labeled, and hybridized to the gene chip. After hybridization and washing, the slides were scanned with the GeneChip GCOS Software (Affymetrix, USA). Raw data extraction and subsequent data processing were performed using the Affymetrix GeneChip Operating Software (Affymetrix, USA).

The random variance model (RVM) *t*-test was applied to filter the differentially expressed genes between LCPD and Control groups, as it can effectively increase the degrees of freedom in cases of small samples. After significance analysis and false discovery rate (FDR) analysis, differentially expressed genes were selected according to their *p*-value threshold and fold change. The threshold set for up-regulated and down-regulated mRNAs/lncRNAs was a fold change >1.2 and a *p*-value < 0.05. The differentially expressed probe sets were imported into Cluster and TreeView (Stanford University) to perform hierarchical cluster analysis (HCA).

### Bioinformatics analysis

Gene Ontology (GO) analysis was applied to explore the function of differentially expressed genes, and to assign the genes to biological processes GO terms according to the annotations. Pathway analysis was applied to find out significant pathways of the differential expression genes according to the Kyoto Encyclopedia of Genes and Genomes (KEGG) database. Two-side Fisher's exact test and χ^2^ test were used to classify the significant pathways. The FDR was calculated to correct the *p*-value, and the threshold of significance was defined by *p*-value < 0.05.

The interaction network of the significant pathways was built according to the interaction among pathways of the KEGG database to find the interaction among the significant pathways directly and systemically. Gene co-expression network was built according to the normalized signal intensity of differentially expressed genes.

### Quantitative real-time quantitative PCR validation

Another 3 LCPD tissues and 3 control tissues were used for qRT-PCR validation. After RNA isolation, qRT-PCRs were performed according to the instructions of the SYBR Premix Ex Taq™ II kit (Takara, Dalian, China.) and carried out in the StepOnePlus real-time PCR system. Each reaction was performed in a final volume of 10 μl containing 0.5 μl PCR Forward Primer (10μM), 0.5 μl PCR Reverse Primer (10μM), 2 μl cDNA, 5 μl 2 × Master Mix and 2 μl RNase-free water. The conditions for qRT-PCR were as follows: 10 min at 95 °C, followed by 40 cycles of 95 °C for 15 s and 60 °C for 30 s. β-actin was used as a reference. Results were harvested in three independent wells. For quantitative results, the relative expression level of each gene was calculated using the 2-ΔΔCt method. Student's *t*-tests were applied, and *p*-value < 0.05 was considered statistically significant (*n* = 3 samples from control vs. LCPD groups). The values were expressed as means ± SD. The primers are listed in Table 3.

**Table 3:** Primers used for PCR.

**Primer name**	**Sequences**
ILK_F	AAGGTGCTGAAGGTTCGAGA
ILK_R	ATACGGCATCCAGTGTGTGA
VCL_F	ATCTCAGGGTCTGGATGTGC
VCL_R	GCACCTCGAATCTGCTCTTC
RRAS_F	CATTAACGACCGGCAGAGTT
RRAS_R	CTCCAGATCTGCCTTGTTCC
TLN1_F	AATCAGGCAGCCACAGAACT
TLN1_R	ATGCCCTTCAAGTTGGACAC
ITGA5_F	CGCTCTCAACTTCTCCTTGG
ITGA5_R	GCAAGATCTGAGCCTTGTCC
PDGFRB_F	GTGGTGATCTCAGCCATCCT
PDGFRB_R	CCTTCCATCGGATCTCGTAA
n335645_F	CCACAAAGTGGATTGCACAC
n335645_R	AGGTGGAATGCGTGTGTTTA
n335724_R	AGTGGCAGTAGCCCAGAAGA
n339347_F	CCACGGAGCAGGTAACATTT
n339347_R	GCTGTCCTTAGGCAGACTGG
n339477_F	GGCATCAGGTTCTCTCAGGA

### Statistical analysis

Statistical evaluations were performed using GraphPad Prism 7. Data are presented as means ± SD. The differences between the two groups were determined by Student's *t*-test, and *p* < 0.05 was considered statistically significant (^*^p < 0.05, ^**^p < 0.01). Each experiment was repeated to produce three biological replicates (*n* = 3 samples from control vs. LCPD groups).

## Results

### Differentially expressed lncRNA and mRNA profiles by microarray

**Figure 1:**
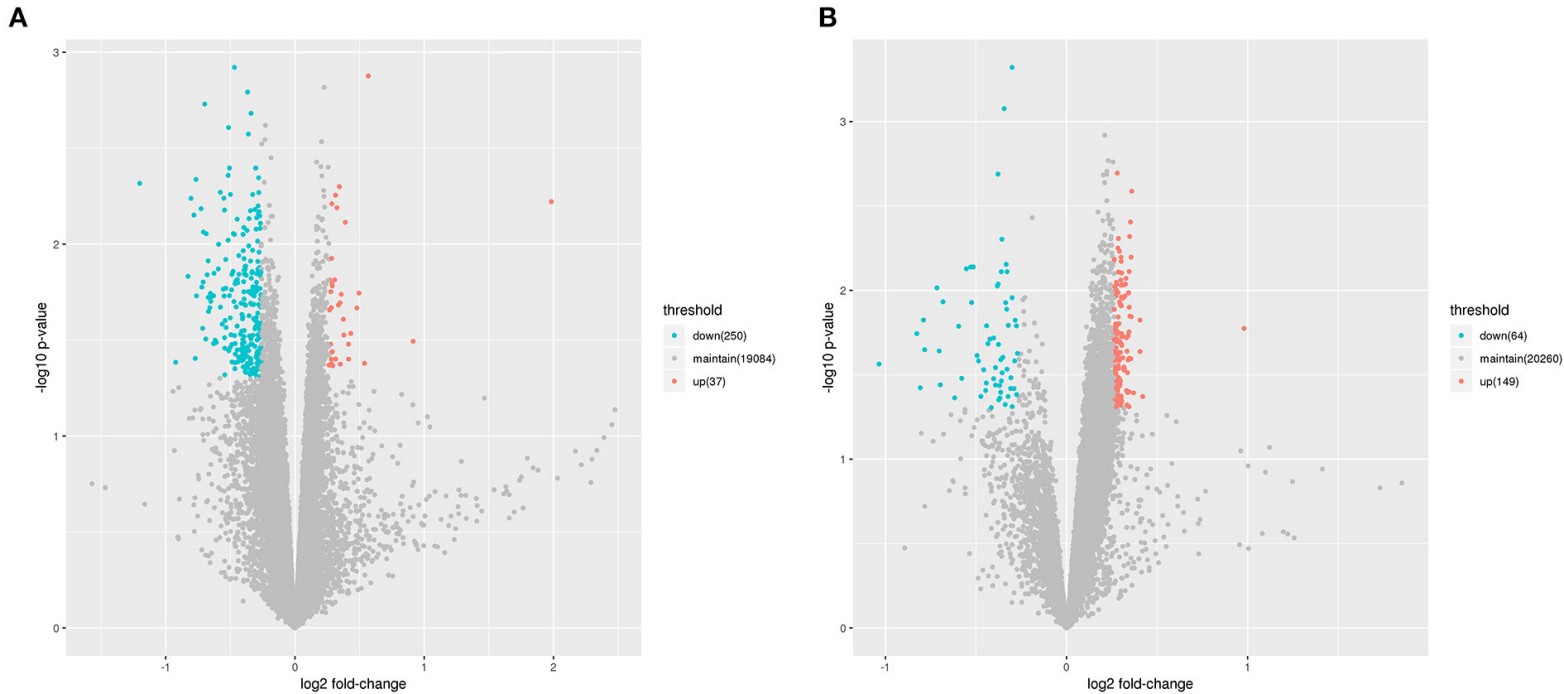
The Volcano Plot showing the variation of lncRNA **(A)** and mRNA **(B)** expressions. The red points indicate up-regulated genes (Fold change > 1.2), and green points indicate down-regulated genes (Fold change < 0.83).

**Figure 2:**
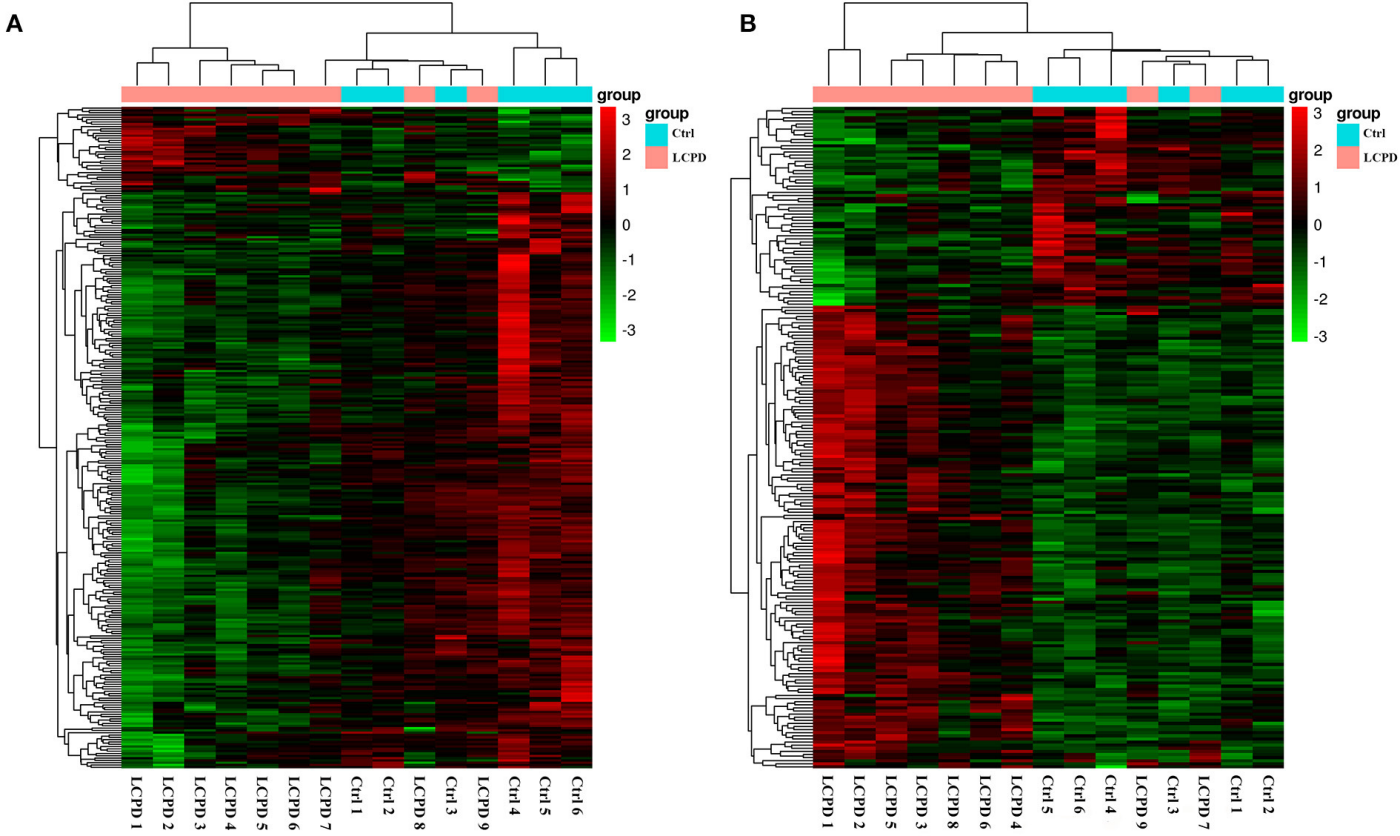
Hierarchical clustering analysis showed the differentially expressed mRNAs **(A)** and lncRNAs **(B)**. The abscissa represents the samples, and the ordinate represents the differentially expressed genes. Red color represents the differentially expressed genes with high expression value, while green color with low expression value.

As mentioned above, the lncRNA expression profiles and the potential regulatory role in the etiology/pathogenesis of LCPD have not been investigated. In order to investigate the potential role of lncRNA in LCPD, a genome-wide analysis was performed to profile the differentially expressed lncRNA and mRNA in LCPD. According to the microarray data, a total of 213 lncRNAs and 287 mRNAs (Fold change > 1.2, *p* < 0.05) were differentially expressed in LCPD periosteum tissues compared with control groups (Supplementary Files 1, 2). Among them, 149 lncRNAs and 37 mRNAs were up-regulated, and 64 lncRNAs and 250 mRNAs were down-regulated (Volcano plots, Figures 1A,B). Hierarchical clustering was used to analyze the gene expression patterns between the groups, and results showed that the lncRNA and mRNA expression profiles were distinguishable between them (heatmap, Figures 2A,B).

### GO and KEGG pathway analysis

**Figure 3:**
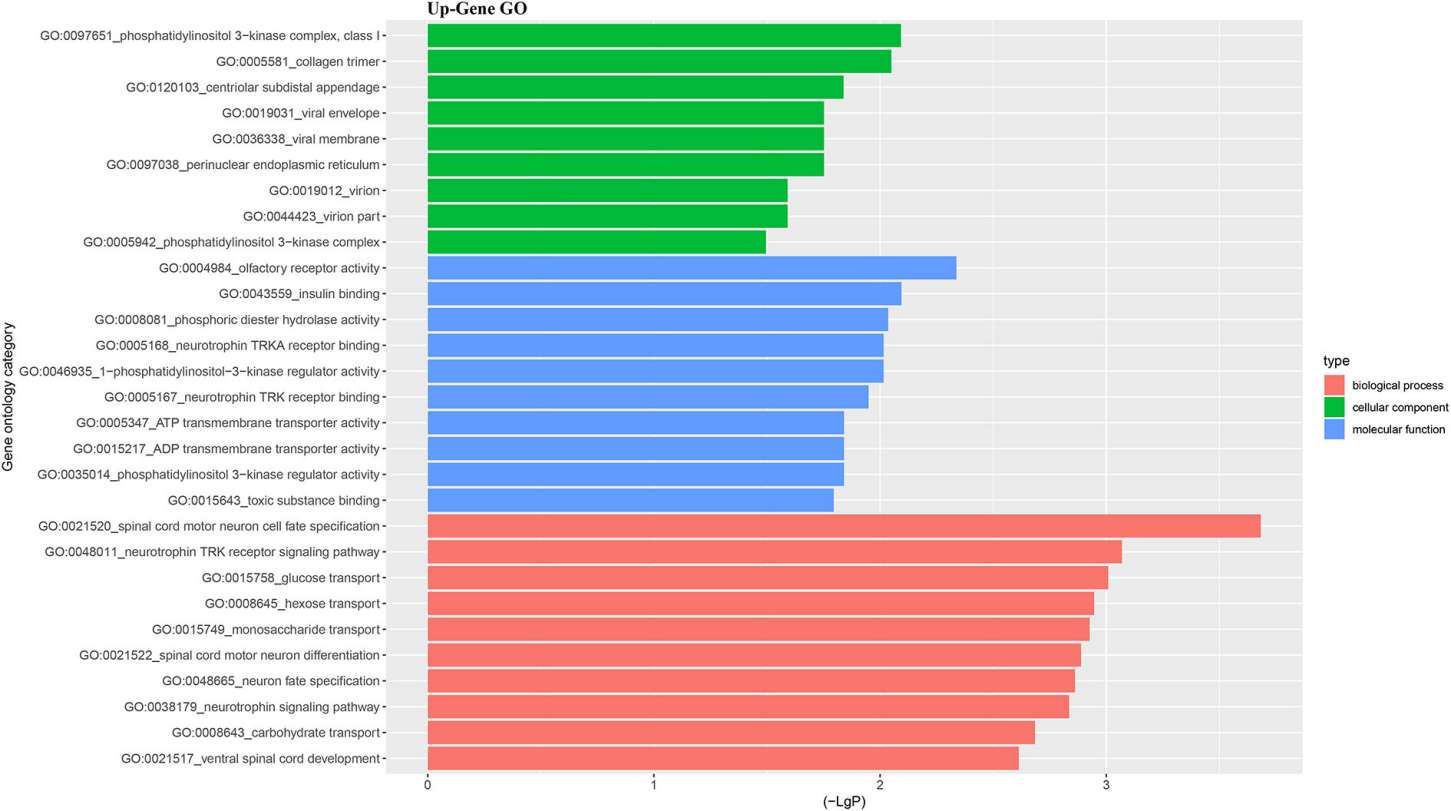
Top 10 up-regulated GO terms of differentially expressed mRNAs which covering biological process, cellular component and molecular function, respectively.

**Figure 4:**
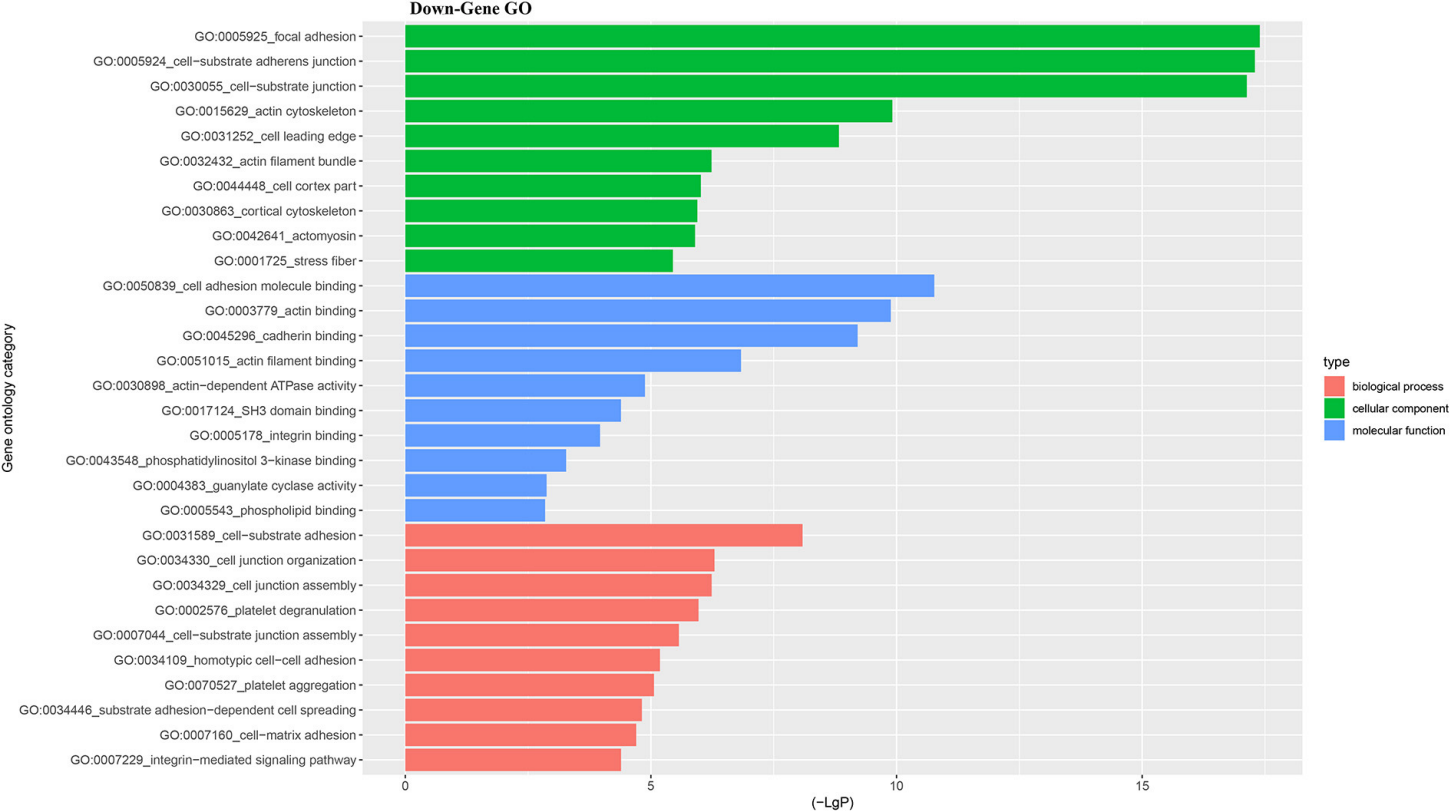
Top 10 down-regulated GO terms of differentially expressed mRNAs which covering biological process, cellular component and molecular function, respectively.

**Figure 5:**
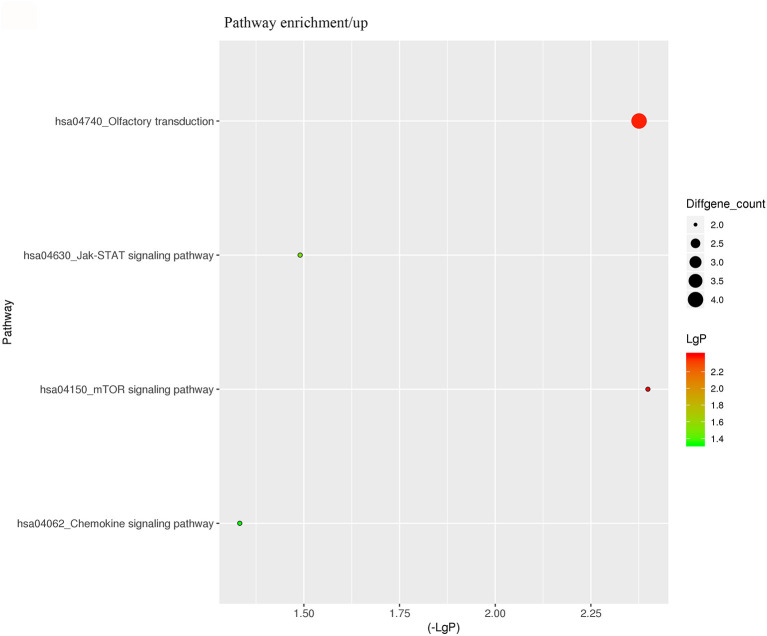
KEGG Pathway analysis of differentially expressed genes based on the KEGG database. And KEGG pathways enrichments of up-regulated genes. The x-axis represents the negative logarithm of the *p*-value (-Lg P). The larger the value, the greater the significant difference of this pathway. The size of the bubble represents the number of differentially expressed genes in the pathway.

**Figure 6:**
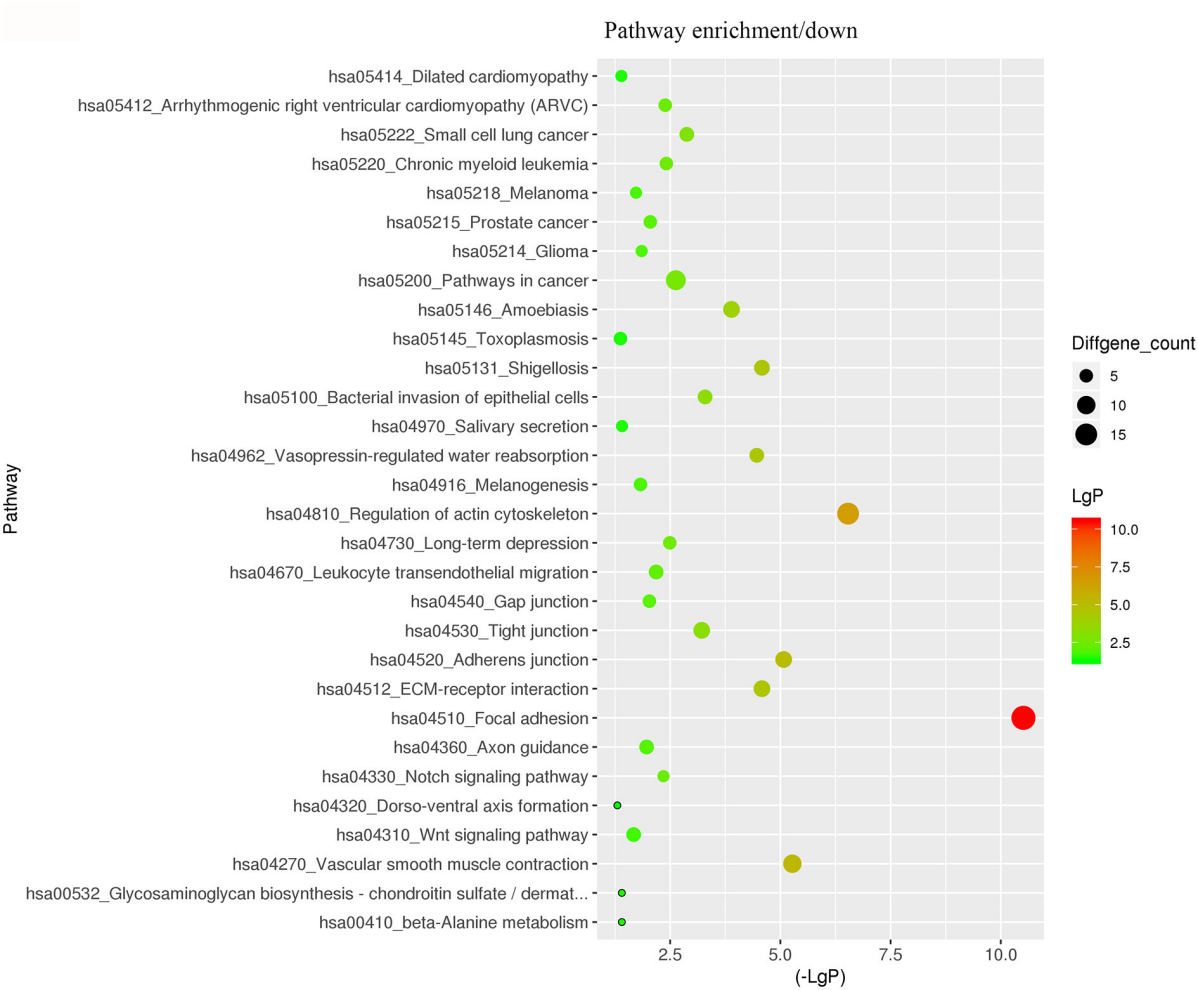
KEGG Pathway analysis of differentially expressed genes based on the KEGG database. And KEGG pathways enrichments of down-regulated genes. The x-axis represents the negative logarithm of the *p*-value (-Lg P). The larger the value, the greater the significant difference of this pathway. The size of the bubble represents the number of differentially expressed genes in the pathway.

For better understanding the relations and biological functions of the differentially expressed genes, GO analysis was performed according to GO terms which included biological processes, cellular components, and molecular functions (Supplementary Files 3, 4). The top 10 up- and down-regulated terms enriched in each of the three categories are shown in Figures 3, 4. KEGG Pathway enrichment was also performed based on KEGG database to reveal the pathway that might be associated with LCPD, including 4 pathways involving up-regulated genes, and 30 pathways involving down-regulated genes (Figures 5, 6, Supplementary Files 3, 4).

**Figure 7:**
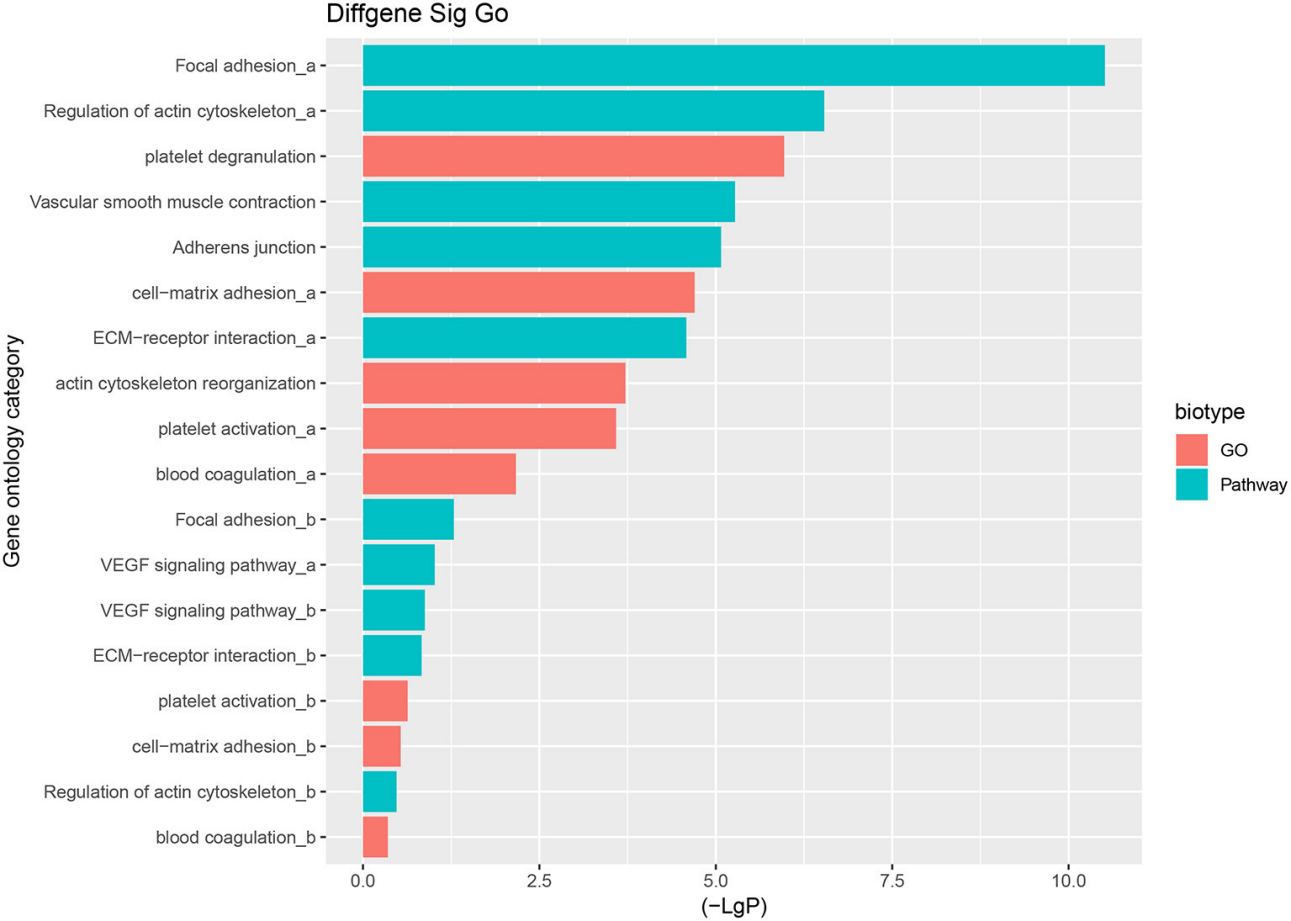
Selected GO terms and Pathways which are involved in coagulation dysfunction, or abnormalities in vascular structure and function based on GO and KEGG pathway analysis (a represents up-regulated GO terms or Pathways, b represents down-regulated GO terms or Pathways).

The predominant opinion is that LCPD disease is multifactorial and may be caused by a combination of genetic and environmental factors. A possible explanation is that genetic factors confer susceptibility to the disruption of the blood supply to the capital femoral epiphysis (2). Therefore, the authors team speculated that disruption of blood supply of femoral head due to coagulation dysfunction, abnormalities in vascular structure and function, or other reasons, might be the critical pathological event in LCPD. Thus, based on the GO analysis and KEGG analysis, we mainly focused on the GO terms and Pathways which were involved in coagulation dysfunction, or abnormalities in vascular structure and function (Figure 7). And through the analysis of the above disease-related significant functions and signaling pathways, we finally found 14 differentially expressed mRNAs possibly involved in the pathogenesis of LCPD (Table 4).

**Table 4:** Concerned differentially expressed mRNAs.

**mRNAs**	**Chromosome**	**Fold change**	**Regulation**	
ACTA2	chr19	1.574014	down	
ACTN1	chr9	1.534254	down	
CD44	chr11	1.573085	down	
COL4A1	chrX	1.613343	down	
CSRP1	chr20	1.695529	down	
FLNA	chr5	1.774544	down	
ILK	chr11	1.50853	down	
ITGA5	chr1	1.895988	down	
MRVI1	chr10	1.590007	down	
MYL9	chr10	1.636164	down	
PDGFRB	chr11	1.647416	down	
RRAS	chr13	1.609621	down	
TLN1	chr14	1.505757	down	
VCL	chr12	1.592037	down	

### Construction of lncRNA-mRNA interaction network

**Figure 8:**
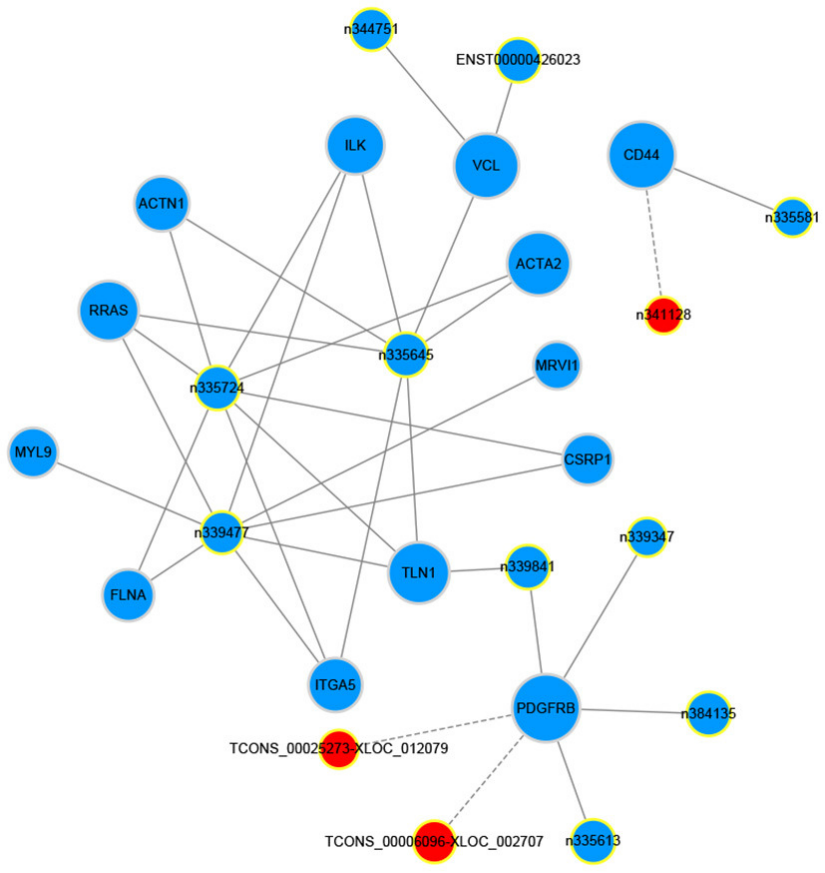
Co-expression network of our concerned differentially expressed mRNAs and lncRNAs. The dots in the figure represent mRNAs, and the yellow circled dots represent lnRNAs (The red indicates up-regulated lncRNAs, and blue, down-regulated). The line represents the regulatory relationship between the two genes (Solid line represents positive regulation, and the dashed line represents negative regulation). The size of the circle represents the ability of a lncRNA/mRNA to interact with other genes, and this ability is quantified by degree. The greater the degree, the more genes that interact with this lncRNA/mRNA.

The gene co-expression network can reveal the relationship between mRNAs and lncRNAs. So, we constructed a co-expression network between the concerned differentially expressed mRNAs and all the differentially expressed lncRNAs based on their expression values, analyzing the possible interacting relationships of these mRNAs and lncRNAs. Through the analysis of the interaction between the concerned differentially expressed mRNAs and lncRNAs, we finally found 13 lncRNAs which might be implicated in the pathogenesis of LCPD (Table 5). In network analysis, the degree was the most important measure of an mRNA or lncRNA centrality within a network. Degree centrality was defined as the link numbers one node has to the other. From the co-expression network, we could find that lncRNA n335645, n335724, n339477 were at the cores. These three lncRNAs could interact with most of our concerned mRNAs and positively regulate their expression (Figure 8). Similarly, one mRNA could also be regulated by multiple lncRNAs, such as PDGFRB (Figure 8).

**Table 5:** Concerned differentially expressed lncRNAs screened through analysis of mRNA-lncRNA co-expression network.

**mRNAs**	**Chromosome**	**Fold change**	**Regulation**
ENST00000426023	chr8	2.049241	down
n335581	chr6	1.312267	down
n335613	chr7	1.62087	down
n335645	chr16	1.728929	down
n335724	chr19	1.642049	down
n339347	chr3	1.340619	down
n339477	chr3	1.773601	down
n339841	chr18	1.400418	down
n344751	chrX	1.255546	down
n384135	chr22	1.275743	down
n341128	chr11	1.265929	up
TCONS_00006096-XLOC_002707	chr3	1.260406	up
TCONS_00025273-XLOC_012079	chr17	1.208068	up

### Validation of deregulated lncRNAs and mRNAs with qRT-PCR

**Figure 9:**
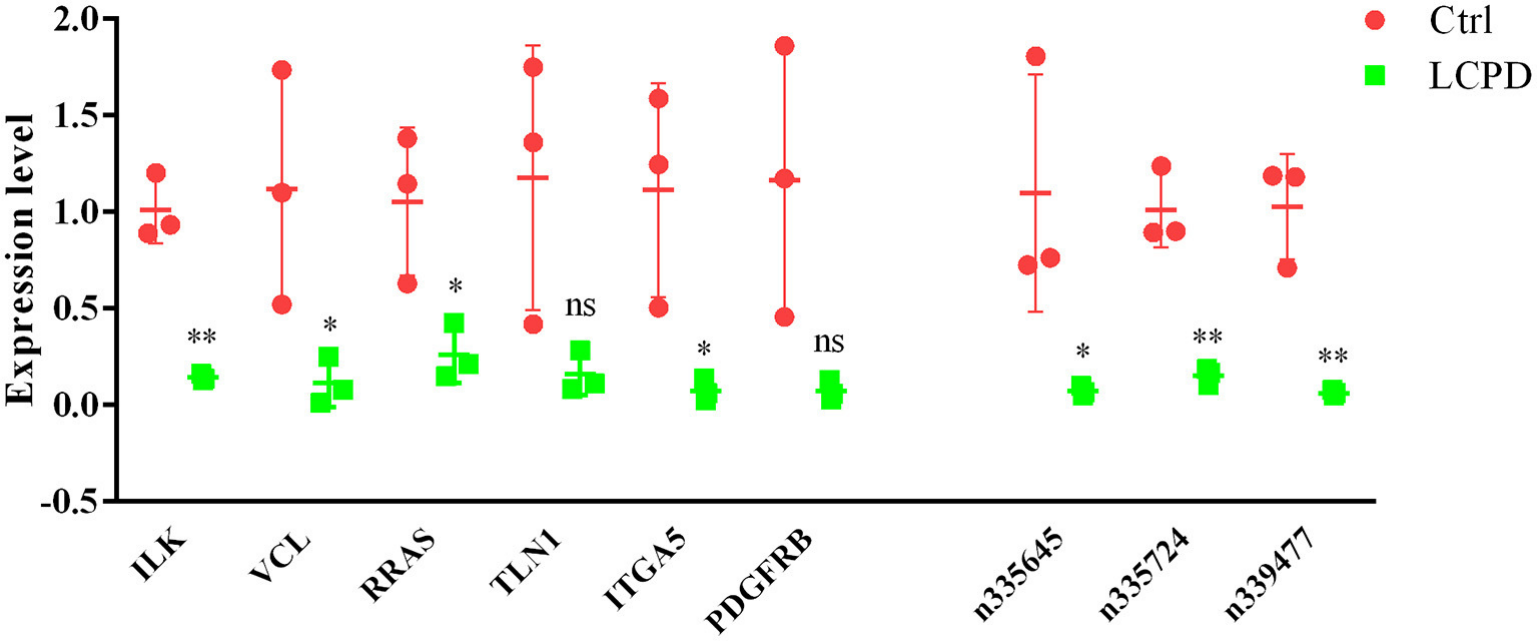
The expression of 3 selected LncRNAs and 6 mRNAs were validated by RT-qPCR in tissues from LCPD group (*n* = 3) and the control group (*n* = 3). Data are presented as means ± SD, and relative gene expression level were normalized to the expression of GAPDH (*P < 0.05, **P < 0.01). ns, no significance.

To further confirm the reliability of the data of differentially expressed genes, the expression of 6 screened mRNAs (ILK, VCL, RRAS, TLN1, ITGA5, and PDGFRB) and 3 lncRNAs (n335645, n335724, and n339477) were detected by qRT-PCR in other 3 independent LCPD and 3 control tissues. From the co-expression network, we could find that lncRNA n335645, n335724, n339477 were at the cores, and the six mRNAs have at least 3 links to the other gene. The results were consistent with our microarray data (Figure 9). The expression of all the selected genes were significantly lower in patients with LCPD (*p* < 0.05) except for TLN1 and PDGFRB (*p* > 0.05). The inconsistency of results of TLN1 and PDGFRB between qRT-PCR and microarray may be due to the individual differences in periosteum from different patients and the small number of samples used for qRT-PCR.

## Discussion

LCPD is a common orthopedic disorder and is characterized by idiopathic osteonecrosis of the femoral head during childhood. Although the condition is self-limiting, it can cause the deformation of the femoral head and hip joint, leading to a limited range of motion and increased risks for secondary arthritic changes. Although it has already been a century that the disease was described first, the etiology of LCPD stills remains unknown (2). A prevailing view is that LCPD is a multifactorial disease caused by a combination of genetic and environmental factors. The disruption of blood supply to the femoral head appears to be the critical pathological event. Genetic factors impart “susceptibility” to the disruption of the blood supply to the femoral head, whereas environmental factors trigger the disease (4–8). In this study, we conducted a genome-wide analysis of lncRNA and mRNA expression profiles in periosteum from LCPD patients and control patients for the first time to screen altered genes that involved in coagulation dysfunction and abnormalities in vascular structure and function which might contribute to the disruption of blood supply for better understanding the pathogenesis of LCPD. And, finally we identified several abnormally expressed mRNAs in periosteum from LCPD patients and constructed the interaction network between the selected mRNAs and differentially expressed lncRNAs.

According to the results of GO and KEGG analysis, we selected several GO terms and pathways (focal adhesion, vascular smooth muscle contraction, VEGF signaling pathway, platelet activation, blood coagulation, and so on) that are known to be associated with abnormalities in vascular structure and function, or coagulation dysfunction, for further analysis. After filtering, we finally found 14 mRNAs that might be involved in the pathogenesis of LCPD and used these genes to construct an mRNA-lncRNA interacting network. And, through the analysis of the interaction network, we finally found 13 differentially expressed lncRNAs which might be implicated in the pathogenesis of LCPD.

As already known, blood supply disruption to the femoral head might be the critical pathological event. Therefore, genetically, abnormal gene expression that can contribute to the interruption of blood supply may be involved in the pathogenic process of LCPD. Integrin-linked kinase (ILK) has been reported to play an essential role in the regulation of angiogenesis, endothelial survival and apoptosis, vasomotor tone, vascular remodeling, osteoblast function, and bone remodeling (31–33). And, angiogenesis ability was impeded when ILK activity was blocked with siRNA or inhibitor (34). Vinculin (VCL) is a component of focal adhesions and adherens junctions and plays a vital role in cell-cell and cell-matrix adhesions, the abnormity of which may lead to vascular dysfunction and vascular disease (35, 36). RRAS is a kind of small GTPase that can affect vessel formation and remodeling by regulating cellular signaling in endothelial cells, pericytes or smooth muscle cells (37). Thus, the dysregulation of these genes may be underlying factors leading to abnormalities of vascular structure or function and contributing to the pathogenesis of LCPD.

LncRNAs have been considered as the clinical diagnostic biomarkers for diseases, and their abnormal expression is usually involved in the pathogenesis of certain diseases (26, 27). Through the interacting analysis between the concerned differentially expressed mRNAs and lncRNAs, several potential disease-related lncRNAs were screened out. According to the co-expression network, lncRNA n335645, n335724, and n339477 were at the center of this network, interacting with most of the concerned differentially expressed mRNAs and positively regulating their expression. In addition, the expression of these three novel lncRNAs were further confirmed by qPCR, and results indicated that the expression of lncRNA n335645, n335724, and n339477 were also decreased in LCPD. As is known, lncRNA can regulate gene expression through various mechanisms. In LCPD patients, decreased expression of certain lncRNA such as lncRNA n335645 may downregulate the expression of correlating mRNA (ILK, VCL, RRAS, or other genes), thus leading to thus impairing of vascular structure or function and leading to disruption of the blood supply to the femoral head in LCPD patients.

**Figure 10:**
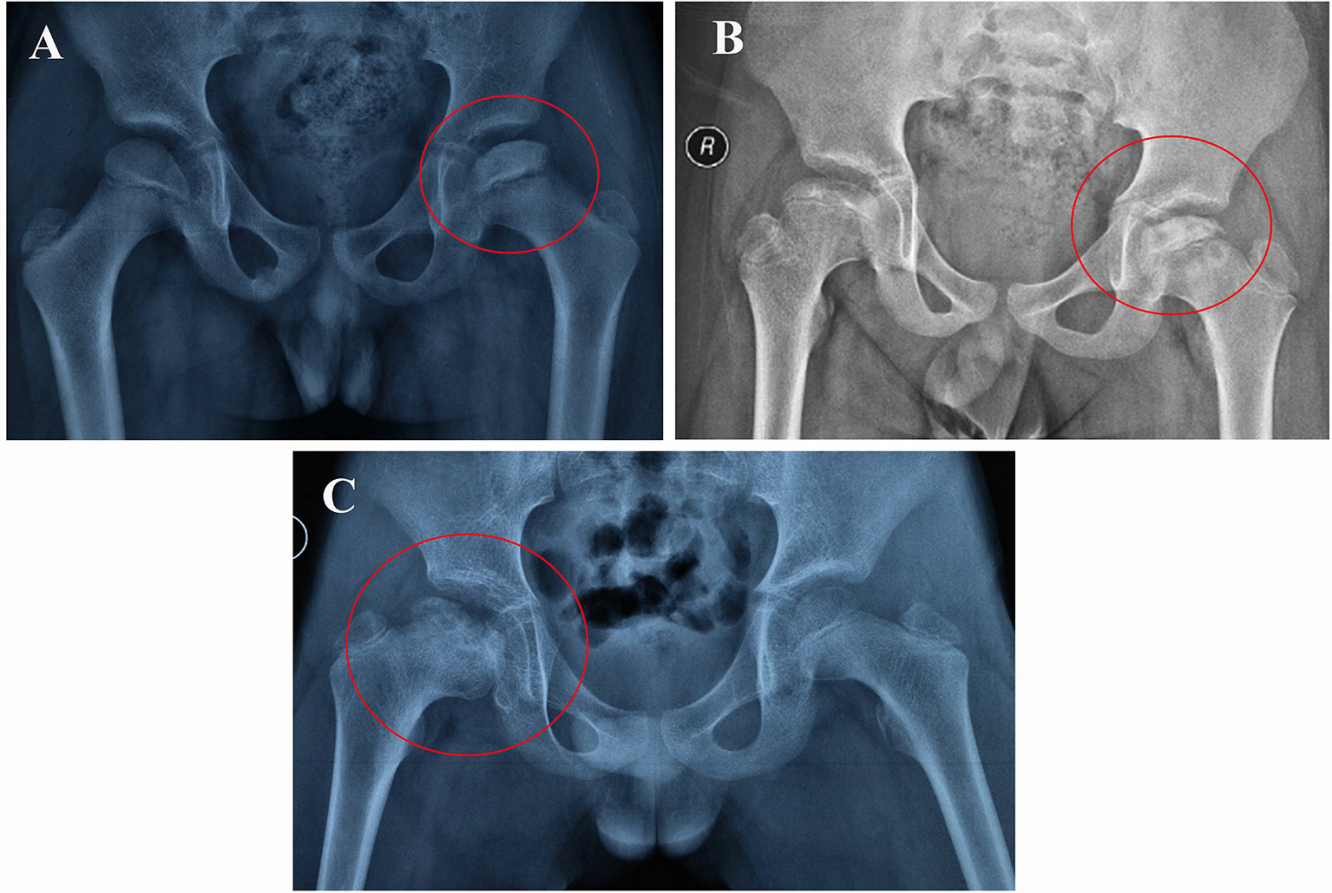
The plain radiographic appearances of the femoral head from different stages of LCPD patients (the red circle). Necrosis stage, 8-year old boy involved left femoral head **(A)**; Fragmentation stage, 9-year old boy involved both hip **(B)**; Reossification stage, 10-year old boy involved right hip **(C)**.

From the radiological point of view, the process of ischemia and subsequent bone regeneration have been divided into several stages (38), including initial or necrosis stage (Figure 10A), fragmentation stage (Figure 10B), reossification stage (Figure 10C), and Final healing stage. However, the symptoms of children with LCPD in the early stages are usually ignored by their parents, so the majority of the children are in the middle and late stages of the disease when come to the outpatient clinic firstly. And the reported incidence of LCPD varies from 0.2 to 19.1 per 100000 (2). Considering the above factors, the sample size of this study was relatively small which is a limitation of our research, and the association of the results with LCPD stages was not analyzed and discussed in this study. In general, surgical treatment is not necessary in patients at early stages, which have full and painless range of motion of the hip and are low risk radiologically regarding the femoral head (38). In authors' hospital, derotation osteotomy of proximal femur combined with pelvic osteotomy are more preferred for the treatment of LCPD who need surgical treatment. During surgery, the femoral head was usually kept completed and if the femoral head cartilage or subchondral bone is sampled, the necrosis and collapse of the femoral head may be aggravated. So, it is difficult to get original tissues of the femoral head. Based on the above considerations, the authors' team collected the periosteum of proximal femur when performing subtrochanteric osteotomy. Periosteum contains a variety of cellular components, such as vascular endothelial cells, osteoblasts, and even stem cells. This is also a limitation of this study, as it is difficult to reflect the actual genetic changes in the tissues of the femoral head.

As blood supply disruption to the femoral head is the initial pathological event, revascularization of bone is also the critical event in osteonecrosis repair process. According to the results of this study, upregulating the expression of these abnormal genes may potentially ameliorate the blood supply of the femoral head and accelerate bone repair. Thus, the findings of this study may provide new sights for future studies on molecular mechanisms or even treatment of LCPD. However, the biological function of these lncRNAs and their diagnostic or therapeutic potential in LCPD need more experimental study in the future.

## Conclusions

Through microarray analysis of lncRNA and mRNA expression profiles in tissues from LCPD patients, a co-expression network of disease-related lncRNAs and mRNAs which screened out from our concerned G.O. terms and Pathways was established. The findings of this study may provide new sights for future studies on molecular mechanisms of LCPD.

## Data availability statement

The datasets presented in this study can be found in online repositories. The names of the repository/repositories and accession number(s) can be found in the article/Supplementary material.

## Ethics statement

The studies involving human participants were reviewed and approved by the Ethics Committee of Tongji Medical College, Huazhong University of Science and Technology (IORG No: IORG0003571). Written informed consent to participate in this study was provided by the participants' legal guardian/next of kin.

## Author contributions

SW, RZ, and PH were involved in data collection and formal analysis. XT and SW were responsible for literature search and study design. SW and HZ drafted the manuscript. XT and JL finalized the manuscript. All authors contributed to the article and approved the submitted version.

## Funding

This work was supported by grants from the National Nature Science Foundation of China (81470100).

## Conflict of interest

The authors declare that the research was conducted in the absence of any commercial or financial relationships that could be construed as a potential conflict of interest.

## Publisher's note

All claims expressed in this article are solely those of the authors and do not necessarily represent those of their affiliated organizations, or those of the publisher, the editors and the reviewers. Any product that may be evaluated in this article, or claim that may be made by its manufacturer, is not guaranteed or endorsed by the publisher.

## References

[1] Mazloumi SM Ebrahimzadeh MH Kachooei AR. Evolution in diagnosis and treatment of Legg-Calve-Perthes disease. Arch Bone Jt Surg. (2014) 2:86–92. 25207324 PMC4151449

[2] Ibrahim T Little DG. The pathogenesis and treatment of Legg-Calvé-Perthes disease. JBJS Rev. (2016) 4:e4. 10.2106/JBJS.RVW.15.00063 27509329

[3] Kim HK. Legg-Calve-Perthes disease: etiology, pathogenesis, and biology. J Pediatr Orthop. (2011) 31:S141–6. 10.1097/BPO.0b013e318223b4bd 21857428

[4] Atsumi T Yamano K Muraki M Yoshihara S Kajihara T. The blood supply of the lateral epiphyseal arteries in Perthes' disease. J Bone Joint Surg Br. (2000) 82:392–8. 10.1302/0301-620X.82B3.0820392 10813176

[5] de Camargo FP de Godoy RM Tovo R. Angiography in Perthes' disease. Clin Orthop Relat Res. (1984) 191:216–20. 10.1097/00003086-198412000-00028 6499314

[6] Théron J. Angiography in Legg-Calvé-Perthes disease. Radiology. (1980) 135:81–92. 10.1148/radiology.135.1.7360984 7360984

[7] Conway JJ. A scintigraphic classification of Legg-Calvé-Perthes disease. Semin Nucl Med. (1993) 23:274–95. 10.1016/S0001-2998(05)80109-6 8256137

[8] Lamer S Dorgeret S Khairouni A Mazda K Brillet PY Bacheville E. Femoral head vascularisation in Legg-Calvé-Perthes disease: comparison of dynamic gadolinium-enhanced subtraction MRI with bone scintigraphy. Pediatr Radiol. (2002) 32:580–5. 10.1007/s00247-002-0732-5 12136349

[9] Kim HK Su PH. Development of flattening and apparent fragmentation following ischemic necrosis of the capital femoral epiphysis in a piglet model. J Bone Joint Surg Am. (2002) 84:1329–34. 10.2106/00004623-200208000-00007 12177261

[10] Shapiro F Connolly S Zurakowski D Menezes N Olear E Jimenez M. Femoral head deformation and repair following induction of ischemic necrosis: a histologic and magnetic resonance imaging study in the piglet. J Bone Joint Surg Am. (2009) 91:2903–14. 10.2106/JBJS.H.01464 19952254 PMC2780921

[11] Calvert PT Kernohan JG Sayers DC Catterall A. Effects of vascular occlusion on the femoral head in growing rabbits. Acta Orthop Scand. (1984) 55:526–30. 10.3109/17453678408992952 6507074

[12] Aksoy MC Aksoy DY Haznedaroglu IC Sayinalp N Kirazli S Alpaslan M. Thrombomodulin and GFC levels in Legg-Calve-Perthes disease. Hematology. (2008) 13:324–8. 10.1179/102453308X343509 19055859

[13] Balasa VV Gruppo RA Glueck CJ Wang P Roy DR Wall EJ. Legg-Calve-Perthes disease and thrombophilia. J Bone Joint Surg Am. (2004) 86:2642–7. 10.2106/00004623-200412000-00009 15590848

[14] Vosmaer A Pereira RR Koenderman JS Rosendaal FR Cannegieter SC. Coagulation abnormalities in Legg-Calvé-Perthes disease. J Bone Joint Surg Am. (2010) 92:121–8. 10.2106/JBJS.I.00157 20048104

[15] Perry DC Green DJ Bruce CE Pope D Dangerfield P Platt MJ. Abnormalities of vascular structure and function in children with Perthes disease. Pediatrics. (2012) 130:e126–31. 10.1542/peds.2011-3269 22665417

[16] Liu R Fan L Yin L Wang K Miao W Song Q. Comparative study of serum proteomes in Legg-Calve-Perthes disease. BMC Musculoskelet Disord. (2015) 16:281. 10.1186/s12891-015-0730-z 26438379 PMC4595068

[17] Zhao Y Liao S Lu R Dang H Zhao J Ding X. Endothelial nitric oxide synthase gene polymorphism is associated with Legg-Calvé-Perthes disease. Exp Ther Med. (2016) 11:1913–7. 10.3892/etm.2016.3111 27168827 PMC4840501

[18] Li N Yu J Cao X Wu QY Li WW Li TF. A novel p. Gly630Ser mutation of COL2A1 in a Chinese family with presentations of Legg-Calvé-Perthes disease or avascular necrosis of the femoral head. PLoS ONE. (2014) 9:e100505. 10.1371/journal.pone.0100505 24949742 PMC4065060

[19] Neidel J Zander D Hackenbroch MH. Low plasma levels of insulin-like growth factor I in Perthes' disease. A controlled study of 59 consecutive children. Acta Orthop Scand. (1992) 63:393–8. 10.3109/17453679209154752 1529687

[20] Ismayilov V Aksoy DY Sayinalp N Haznedaroglu IC Aksoy MC. Increased soluble selectins as a reflection of activated platelets and endothelium in Legg-Calve-Perthes disease. J Pediatr Hematol Oncol. (2014) 36:e410–1. 10.1097/MPH.0000000000000203 25000467

[21] Lee JH Zhou L Kwon KS Lee D Park BH Kim JR. Role of leptin in Legg-Calvé-Perthes disease. J Orthop Res. (2013) 31:1605–10. 10.1002/jor.22415 23832827

[22] Liz J Esteller M. lncRNAs and microRNAs with a role in cancer development. Biochim Biophys Acta. (2016) 1859:169–76. 10.1016/j.bbagrm.2015.06.015 26149773

[23] Esteller M. Non-coding RNAs in human disease. Nat Rev Genet. (2011) 12:861–74. 10.1038/nrg3074 22094949

[24] Li LJ Leng RX Fan YG Pan HF Ye DQ. Translation of noncoding RNAs: Focus on lncRNAs, pri-miRNAs, and circRNAs. Exp Cell Res. (2017) 361:1–8. 10.1016/j.yexcr.2017.10.010 29031633

[25] Flynn RA Chang HY. Long noncoding RNAs in cell-fate programming and reprogramming. Cell Stem Cell. (2014) 14:752–61. 10.1016/j.stem.2014.05.014 24905165 PMC4120821

[26] Bhan A Mandal SS. Long noncoding RNAs: emerging stars in gene regulation, epigenetics and human disease. ChemMedChem. (2014) 9:1932–56. 10.1002/cmdc.201300534 24677606

[27] Li X Wu Z Fu X Han W. lncRNAs: insights into their function and mechanics in underlying disorders. Mutat Res Rev Mutat Res. (2014) 762:1–21. 10.1016/j.mrrev.2014.04.002 25485593

[28] Chi Y Wang D Wang J Yu W Yang J. Long non-coding RNA in the pathogenesis of cancers. Cells. (2019) 8:1015. 10.3390/cells8091015 31480503 PMC6770362

[29] Wang Q Yang Q Chen G Du Z Ren M Wang A. LncRNA expression profiling of BMSCs in osteonecrosis of the femoral head associated with increased adipogenic and decreased osteogenic differentiation. Sci Rep. (2018) 8:9127. 10.1038/s41598-018-27501-2 29904151 PMC6002551

[30] Xiang S Li Z Weng X. The role of lncRNA RP11-154D6 in steroid-induced osteonecrosis of the femoral head through BMSC regulation. J Cell Biochem. (2019) 120:18435–45. 10.1002/jcb.29161 31190361

[31] Herranz B Marquez S Guijarro B Aracil E Aicart-Ramos C Rodriguez-Crespo I. Integrin-linked kinase regulates vasomotor function by preventing endothelial nitric oxide synthase uncoupling: role in atherosclerosis. Circ Res. (2012) 110:439–49. 10.1161/CIRCRESAHA.111.253948 22194624

[32] Reventun P Alique M Cuadrado I Márquez S Toro R Zaragoza C. iNOS-derived nitric oxide induces integrin-linked kinase endocytic lysosome-mediated degradation in the vascular endothelium. Arterioscler Thromb Vasc Biol. (2017) 37:1272–81. 10.1161/ATVBAHA.117.309560 28546219

[33] Dejaeger M Böhm AM Dirckx N Devriese J Nefyodova E Cardoen R. Integrin-linked kinase regulates bone formation by controlling cytoskeletal organization and modulating BMP and Wnt signaling in osteoprogenitors. J Bone Miner Res. (2017) 32:2087–102. 10.1002/jbmr.3190 28574598

[34] Li Y Li G Hu X Lin W Sun J Mi L. Integrin-Linked Kinase Senses Hypoxia During Scar Angiogenesis. Int J Low Extrem Wounds. (2016) 15:286–95. 10.1177/1534734616649485 27230895

[35] Bays JL DeMali KA. Vinculin in cell-cell and cell-matrix adhesions. Cell Mol Life Sci. (2017) 74:2999–3009. 10.1007/s00018-017-2511-3 28401269 PMC5501900

[36] Dorland YL Huveneers S. Cell-cell junctional mechanotransduction in endothelial remodeling. Cell Mol Life Sci. (2017) 74:279–92. 10.1007/s00018-016-2325-8 27506620 PMC5219012

[37] Sawada J Li F Komatsu M. R-Ras Inhibits VEGF-Induced p38MAPK Activation and HSP27 Phosphorylation in Endothelial Cells. J Vasc Res. (2015) 52:347–59. 10.1159/000444526 27029009 PMC4842017

[38] Rodríguez-Olivas AO Hernández-Zamora E Reyes-Maldonado E. Legg-Calvé-Perthes disease overview. Orphanet J Rare Dis. (2022) 17:125. 10.1186/s13023-022-02275-z 35292045 PMC8922924

